# Supplementary material for: Synthesis of Silver Nanoparticles Using Extracts from Different Parts of the Paullinia cupana Kunth Plant: Characterization and In Vitro Antimicrobial Activity
Source: Pharmaceuticals (Basel). 2024 Jul 2;17(7):869. doi: 10.3390/ph17070869 (PMC11279972; doi:10.3390/ph17070869)

Supporting Information

Synthesis of silver nanoparticles using extracts from different parts the *Paullinia cupana* Kunth plant: characterization and *in vitro* antimicrobial activity

Alan Kelbis Oliveira Lima[1,8], Lucas Marcelino dos Santos Souza[2], Guilherme Fonseca Reis[3], Alberto Gomes Tavares Junior[4], Victor Hugo Sousa Araújo[4], Lucas Carvalho dos Santos[5], Vitória Regina Pereira da Silva[6], Marlus Chorilli[4], Hugo de Campos Braga[7], Dayane Batista Tada[7], José Antônio de Aquino Ribeiro[8], Clenilson Martins Rodrigues[8], Gerson Nakazato[2] Luís Alexandre Muehlmann[9*], and Mônica Pereira Garcia[1]

Supplementary Figure S1: Chromatograms obtained by UHPLC-HRMS/MS of the aqueous extract of leaves and flowers of *Paullinia cupana*. A) Positive ionization mode; B) Negative ionization mode.

A)

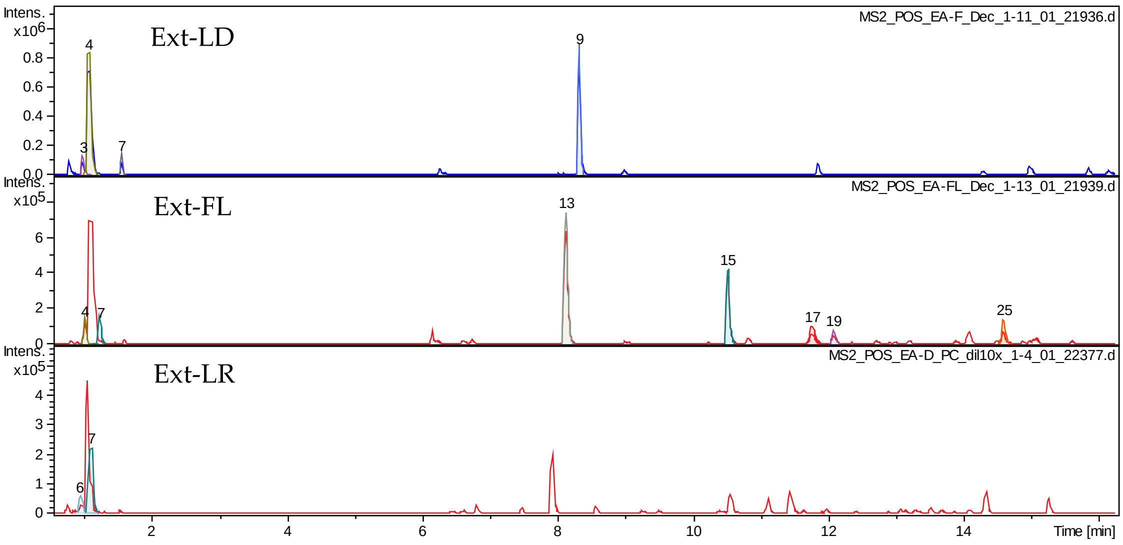

B)

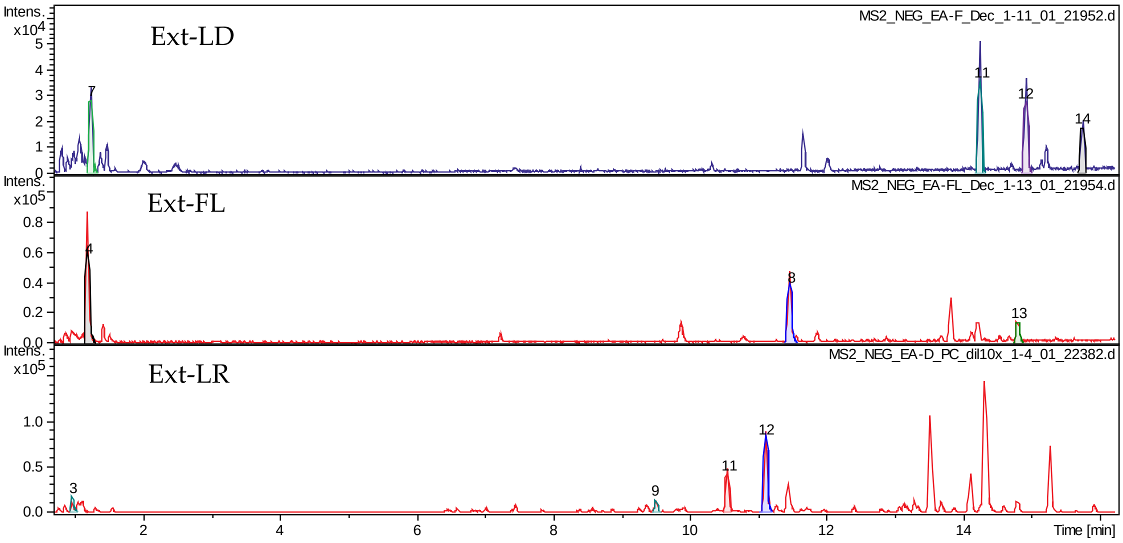

Supplementary Figure S2: Calibration curves of gallic acid (GA) at different concentrations *versus* the absorbances for quantification the total phenol content (760 nm) (A), antioxidant capacity against the DPPH radical (517 nm) (B) and ABTS (734 nm) (C).

A)

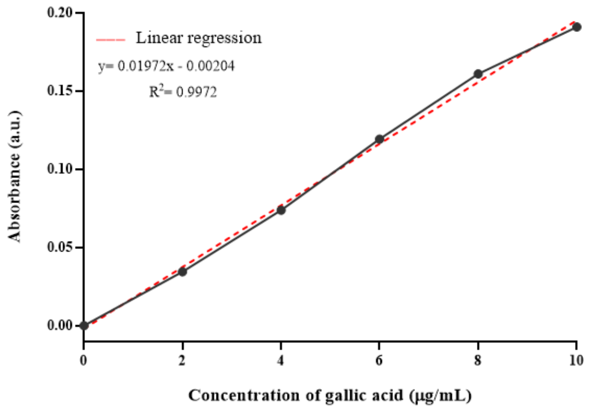

B)

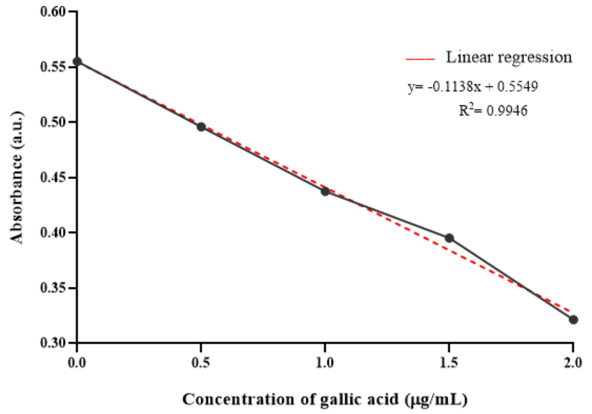

C)

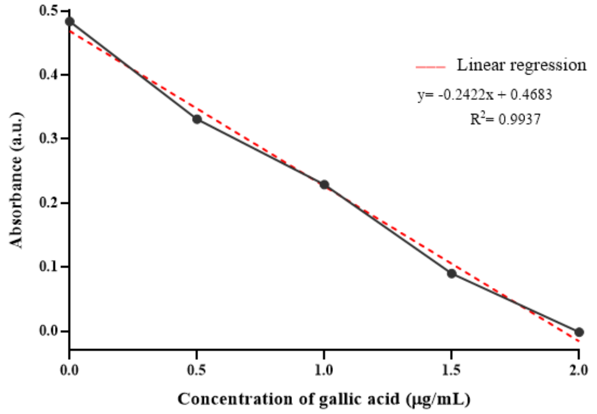

Supplementary Figure S3: Visual record of the colouring of AgNPs-LD (left), AgNPS-FL (centre) and AgNPs-LR (right) after incubation in a water bath at 70 ºC for 180 minutes.

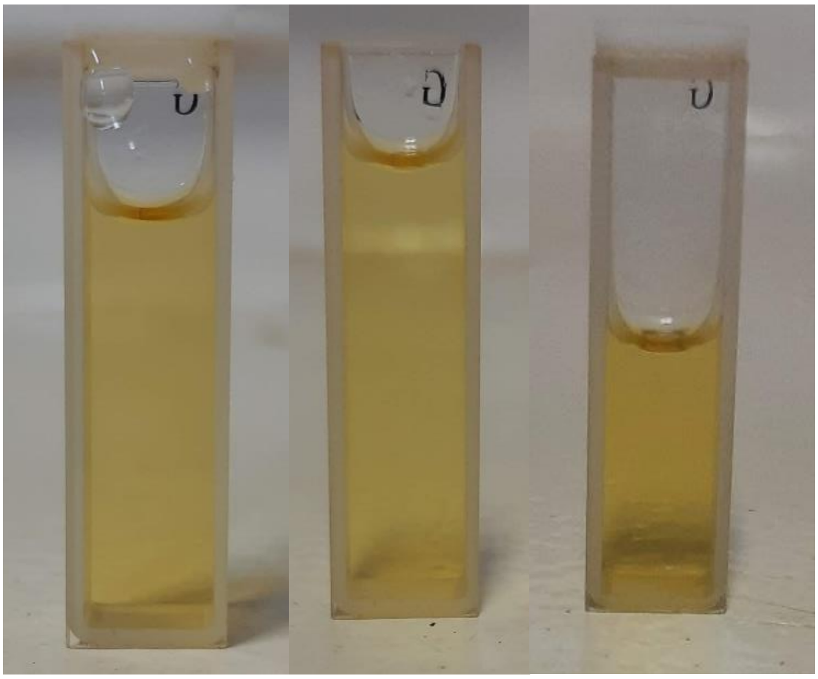

Supplement: Supplementary file 1 [file pharmaceuticals-17-00869-s001.zip › pharmaceuticals-3056011-supplementary.pdf]
